# Leptin, Obesity, and Leptin Resistance: Where Are We 25 Years Later?

**DOI:** 10.3390/nu11112704

**Published:** 2019-11-08

**Authors:** Andrea G. Izquierdo, Ana B. Crujeiras, Felipe F. Casanueva, Marcos C. Carreira

**Affiliations:** 1 Laboratory of Epigenomics in Endocrinology and Nutrition, Epigenomics Unit, Instituto de Investigacion Sanitaria de Santiago (IDIS), Complejo Hospitalario Universitario de Santiago (CHUS/SERGAS), 15706 Santiago de Compostela, Spain; andrea.gonzalez.izquierdo@hotmail.com (A.G.I.); anabelencrujeiras@hotmail.com (A.B.C.); 2 CIBER de Fisiopatologia de la Obesidad y Nutricion (CIBERobn), Instituto Salud Carlos III, 28029 Madrid, Spain; 3 Laboratory of Molecular Endocrinology, Instituto de Investigacion Sanitaria de Santiago (IDIS), Complejo Hospitalario Universitario de Santiago (CHUS), 15706 Santiago de Compostela, Spain; 4 Molecular Endocrinolgy, Universidad de Santiago de Compostela (USC), 15706 Santiago de Compostela, Spain

**Keywords:** leptin, obesity, leptin resistance, blood brain barrier

## Abstract

Leptin, a hormone that is capable of effectively reducing food intake and body weight, was initially considered for use in the treatment of obesity. However, obese subjects have since been found to have high levels of circulating leptin and to be insensitive to the exogenous administration of leptin. The inability of leptin to exert its anorexigenic effects in obese individuals, and therefore, the lack of clinical utility of leptin in obesity, is defined as leptin resistance. This phenomenon has not yet been adequately characterized. Elucidation of the molecular mechanisms underlying leptin resistance is of vital importance for the application of leptin as an effective treatment for obesity. Leptin must cross the blood–brain barrier (BBB) to reach the hypothalamus and exert its anorexigenic functions. The mechanisms involved in leptin transportation across the blood–brain barrier continue to be unclear, thereby preventing the clinical application of leptin in the treatment of obesity. In recent years, new strategies have been developed to recover the response to leptin in obesity. We have summarized these strategies in this review.

## 1. Introduction

Twenty-five years ago, leptin, a 160-kDa hormone produced and secreted by the adipose tissue in direct relation to the amount of body fat, was discovered [1]. Much of the research on leptin performed during the early days focused on its role in regulating energy homeostasis and obesity at the level of the central nervous system. The role of leptin in the regulation of energy homeostasis was demonstrated by observing leptin-deficient patients, who develop hyperphagia and obesity during childhood, and can be aided by leptin replacement therapies that suppress appetite and increase energy expenditure [2]. This attracted a lot of interest toward the clinical use of leptin for the treatment of obesity in humans. However, most obese subjects are not deficient in the leptin gene, and the circulating levels of leptin are elevated compared to those in non-obese subjects. Paradoxically, these obese subjects remain obese, reflecting a state of leptin resistance that leads to the intake of extra calories and prevents sustained weight loss [3,4]. The mechanisms involved in leptin resistance have not been clarified since the discovery of leptin in 1994. Different mechanisms have been suggested, such as elevated levels of C-reactive protein, the downregulation of the leptin-activated signal transduction pathway, or a decrease in histone deacetylase activity [5,6,7]. However, alterations in the transport of leptin to the brain through the blood–brain barrier (BBB), a mechanism that has not been completely deciphered [8], seem to play a fundamental role [9,10,11].

## 2. Blood–Brain Barrier and Obesity

The blood–brain barrier (BBB) is made up of several highly specialized cell types that protect the brain from toxic substances and regulate the passage of macromolecules as well as the bidirectional transport of nutrients and hormones between the blood and brain. Food intake and metabolism are regulated by different hormones, such as leptin, whose circulating levels must be regulated very precisely and are often altered in obesity. These hormones must reach the brain by crossing the BBB through a specific transporter [12]. As many of these transporters are affected by saturation mechanisms, the circulating levels of hormones affect their activity and regulation, and therefore, transporters at the level of the BBB play a critical role in the regulation of metabolism. In addition, obesity can generate pathological changes in the cellular integrity of the BBB, independently of the transporters, which can aggravate the pathological situation at the level of the central nervous system.

Obesity and chronic consumption of a high-fat diet (HFD) produce important changes at the level of the BBB as well as in different regions of the brain, especially in the regions of neuronal populations with high metabolic demands, such as the hippocampus [13,14]. Some studies in rodents have shown that feeding with a HFD produces neuronal loss in the arcuate nucleus and hypothalamus [13], in addition to causing a decrease in the integrity of the BBB because of the loss of tanycytes (specialized ependymal cells in the median eminence) and transporters at the level of the BBB [14].

## 3. Leptin, Brain and Blood-Brain Barrier

Leptin is an adipokine that reflects, at the level of the brain, the degree of adiposity of an organism. To exert this action, it must pass through the BBB through a specific and saturable transporter. Right from early studies, it was postulated that as adiposity increases, serum leptin levels also increase, which can lead to the development of resistance at the level of the BBB transporter [10]. This implies that a lesser amount of leptin will reach the brain, thereby leading to reduced activation of the signaling pathway for body weight regulation. Several studies have shown that obese mice are sensitive to intracerebro-ventricular (ICV), but not subcutaneous or intraperitoneal (IP), or the administration of leptin [15,16], indicating that the lack of leptin activity is due to 35% decrease in BBB permeability [10]. Moreover, the cerebrospinal fluid/serum leptin ratio in obese humans is 4–5 times lower [17,18]. These data suggest that reduced brain access is the source of leptin resistance in obesity and further increase in body weight. Until now, it has been unclear which mechanism allows leptin access to the central nervous system to further exert its effects. With a size of 16-kDa, leptin does not appear likely to use a passive diffusion mechanism, although direct access to the neurons in the mediobasal hypothalamus (MBH) region, which are not protected by the BBB, has been observed [19]. The entry of leptin into the brain is partially saturable [20], which indicates the involvement of a protein transporter. Moreover, leptin transport by tanycytes in the MHB requires the presence of the leptin receptor (OBR) [21] as well as the short isoforms of the receptor (OBRa and OBRc) [22,23,24,25,26], which are highly expressed in the BBB. The loss of OBR isoforms reduces the amount of leptin in the brain of mice [26]. Interestingly, the decrease in the passage of leptin through the BBB does not appear to be due to the loss of leptin transporters [27,28]. The molecular mechanism involved in this effect is unknown. Considering that leptin is transported through the BBB by the leptin receptor, which is, therefore, subject to the regulatory mechanisms of the membrane receptors, it is expected that the high levels of circulating leptin could activate the mechanisms of desensitization and downregulation, causing the degradation of these receptors. Leptin resistance at the BBB has been attributed to receptor saturation effects exerted by excess leptin or reversible inhibition caused by circulating factors such as triglycerides [9]. It has been described that at the physiological concentrations of circulating leptin, this transporter works at 50% saturation [29], which suggests that leptin plays its role as a regulator of body weight within very defined and narrow concentration ranges. In addition, with progressing obesity, a phenomenon of double-level resistance is observed in the BBB and in the leptin receptor in the arcuate nucleus [10,16,30].

**Figure 1:**
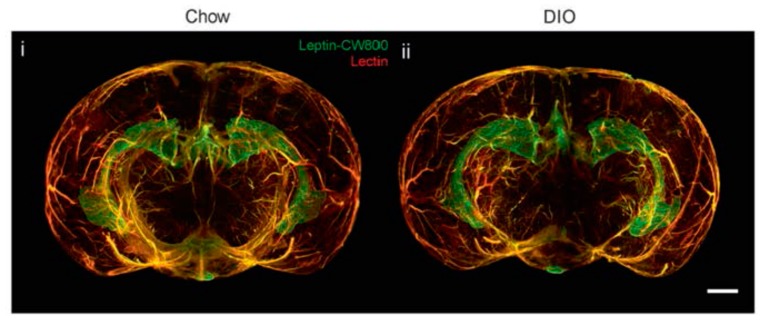
Three-dimensional reconstruction of the brain, indicating the accumulation of fluorescent leptin (leptin-CW800) in the median eminence (ME) and choroid plexus (CP) of lean (Chow, standard diet) (i) and diet-induced obesity (DIO) (ii) mice. From [34], visit http://creativecommons.org/licenses/by/4.0/.

However, new studies have been questioning the proclaimed decrease in leptin transport through the BBB in obese individuals, thereby opening up to the idea of leptin resistance unrelated to transportation through the BBB. Initial studies have shown that the ICV administration of leptin has no effect on food intake or weight loss in diet-induced obesity (DIO) mice [11]. In addition, the use of leptin receptor antagonists in DIO mice indicates that the endogenous leptin remains functional [31], a fact that has also been observed in humans [32]. Unchanged leptin–BBB transport kinetics has also been observed in DIO mice [33]. A recent study investigated the transportation of leptin through the BBB in obese mice by using a novel and interesting visualization technique based on fluorescently labeled leptin and light-sheet fluorescence microscopy [34]. In this crisp article, no differences in leptin accumulation were noted between obese and lean mice in different parts of the brain (Figure 1). In addition, weight loss in these animals by caloric restriction or pharmacological intervention produces an increase in the expression of leptin and its receptor in some parts of the brain. This suggests that in a state of obesity, leptin accumulation is maintained in the key areas of the brain involved in metabolism and weight control. 

During the last 25 years, leptin resistance, as observed in obesity, has been thought to be primarily due to the loss of the capacity of leptin to cross the BBB, chiefly by means of its specific transporter, but considering previously published data, the molecular mechanisms implicated in leptin resistance as well as the mechanism by which the brain pulls up leptin from the systemic circulation are poorly understood.

## 4. Is It Possible to Use Leptin for the Treatment of Obesity?

The weight loss achieved with caloric restriction-based diets, lifestyle modifications, and/or rarely used pharmacological treatments against obesity indicate a recovery of leptin sensitivity, which could be used to maintain body weight.

**Figure 2:**
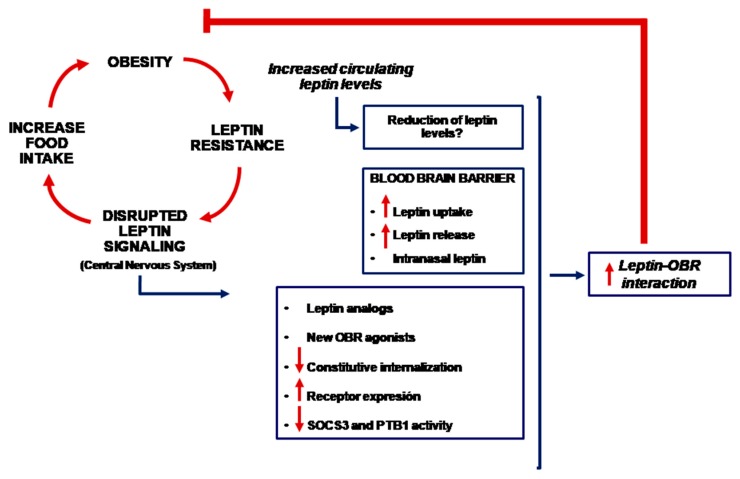
Schematic representation of different anti-obesity therapies based on the use of leptin (OBR = leptin receptor).

Irrespective of the mechanism(s) responsible for leptin resistance, it is plausible to use leptin for weight loss if the leptin receptor and the underlying intracellular signaling pathway are specifically activated in the corresponding parts of the brain. Different methodological approaches have been used (Figure 2).

One of the most intuitive approaches is to increase the passage of leptin through the BBB. It is important to point out that many strategies used to increase the passage of substances through the BBB in different pathological conditions consider that the BBB maintains its physiological properties; however, the pathological condition itself can modify the integrity of the BBB, promoting the failure of these therapies. Therefore, it is necessary that therapies based on an increase in the passage of leptin through the BBB ensure the integrity of the BBB. Strategies that improve the passage of leptin to the brain include the development of modifications in the structure of leptin or leptin analogues as well as the development of new leptin receptor agonists with increased BBB permeability. One of the most widely used modifications in the development of targeted therapeutic agents in the brain is the addition of hydrophilic polyethylene glycol (PEG)-containing polymers. However, PEG-modified leptin is unable to pass through the BBB and thereby reduce body weight in humans [35,36,37]. Other strategies based on the addition of a glucidic residue, such as leptin fused with a trans-activating transcriptional activator Tat (Tat-Leptin) or pluronic, have shown an increase in BBB transportation in DIO mice [38,39,40,41]. In addition, new techniques such as PASylation of leptin, which aim to prolong its half-life, can increase the effectiveness of leptin [42].

Endogenous leptin is a molecule that is not very stable in vivo and has a short half-life, which means that it is less useful in the case of leptin resistance. Therefore, the use of synthetic molecules similar to leptin, which are more stable, may aid in the activation of the OBR. Leptin-related analogs such as 22–56, 57–92, 93–105, and 116–130 are capable of mimicking the interaction and activation of the OBR in order to improve their anti-obesity effects, albeit with relatively limited success [43,44]. OB3 is a synthetic leptin agonist containing the C-terminal aminoacidic residues 116 ≥ 122. This peptide crosses the BBB through an independent mechanism of the OBRb, reaching a higher concentration in the central nervous system compared to leptin, and reducing food intake and body weight in an obesity model of OBRb-deficient *db*/*db* mice [45]. In addition, it regulates energy balance, glycemia, and insulin sensitivity in CB57/BL6 obese mice [46].

Several studies have shown that conventional leptin replacement therapies in obese subjects have very modest effects. To this point, several studies have proposed combinatorial therapies of the different hormones involved in energy regulation to act upon various mechanisms of action and avoid compensatory mechanisms. In leptin-resistant rats, the combination of amylin, a 37-amino acid-long anorexigenic hormone, with leptin results in the greater inhibition of food intake and loss in body weight, when compared to leptin monotherapy, as well as improved metabolism in the long term [47,48,49,50,51,52,53,54]. In humans, a combination of pramlintide acetate (a synthetic analog of amylin) and metreleptin (a methionyl form of leptin) has been used, which caused more weight loss compared to that observed individually with these compounds [55,56]. However, this therapeutic strategy was suspended because of the development of anti-metreleptin antibodies.

Cholecystokinin (CCK) and glucagon-like peptide (GLP-1) and their analogues are other molecules that can be used in combination therapies with leptin. The subcutaneous administration of CCK, amylin, and leptin caused a remarkable reduction in food intake, body weight, and adiposity in DIO mice [57]. The use of leptin and exendin-4, a natural ligand of the GLP-1 receptor, led to the recovery of leptin sensitivity in DIO mice undergoing weight loss [35]. Fibroblast growth factor 21 (FGF21) has also been used as a leptin co-treatment to counteract leptin resistance [35]. It is noteworthy that in some of these studies, the loss of body weight was found to be insufficient to regain sensitivity to leptin, thus indicating the need to use combined strategies with two or more hormones to exert significant and lasting effects on weight loss [35,58]. Leptin-enhancing effects have also been observed in its co-administration with cluterin, a ligand for low-density lipoprotein (LDL) receptor-related protein-2 (LRP2) [59]. Other animal studies have been reported, wherein leptin treatment with insulin has been shown to promote browning of the white adipose tissue [60], and drugs that activate 5-hydroxytryptamine (5-HT) 2C receptors, such as meta-chlorophenylpiperazine, might exert an additive effect on weight loss [61].

The ligand–receptor interaction is important for a variety of biological functions as well as in pharmacological development. The ligand-centered approach is one available approach, while another approach is to focus efforts on the receptor as well as on the subsequently activated intracellular signaling pathway. However, the OBR presents particular characteristics based on its aminoacidic sequence, which accord it particular properties that further regulate the presence of receptors and their activity at the level of the plasma membrane. The OBR exhibits a high degree of constitutive internalization in the absence of interaction with leptin [62,63,64,65,66,67,68]. This property can be attributed to the presence of two lysine residues in the intracellular region of the OBR. On the other hand, the receptors that are internalized after interaction with leptin following the classic desensitization processes have a low recycling rate. In addition, a substantial part of the receptors that are synthesized de novo is retained in the trans-Golgi network [63]. All these incidents result in the reduced expression (5–25%) of the leptin receptor in the plasma membrane [69], thereby naturally reducing leptin sensitivity and intracellular signaling. This could be related to leptin resistance in obesity and could be one of the causes of the limited effects exerted by leptin in anti-obesity therapies. Therefore, therapies that mobilize the OBR from intracellular pools and allow for increased OBR on the cell surface might be useful in achieving greater sensitivity to leptin in obesity. Basic studies have been performed in this direction, identifying ubiquitin ligase RNF41 (a protein encoded by the *db* gene, formed by an alternative splicing, namely, endospanin), and LRP2, which for allow the enhanced presence of OBR on the cell surface and/or activation of key proteins such as STAT3 in the leptin-activated intracellular signaling pathway [70,71,72,73]. These need to be studied in further detail in the context of obesity and leptin resistance.

Once OBR is activated, the leptin-mediated signaling pathway comes into action. In the OBR-leptin system, two proteins are primarily responsible for managing leptin-activated signaling, which are important in terms of response regulation and are therefore potential therapeutic candidates. These proteins are suppressors of cytokine signaling-3 (SOCS3) and protein tyrosine phosphatase-1B (PTP1B). Animal models with SOCS3-specific deletion or modification in the OBR region that alter interactions with SOCS3 have shown increased sensitivity to leptin, and decreased food intake, weight loss, and resistance to DIO [74,75,76,77,78]. However, no specific SOCS3 inhibitors have been developed. On the other hand, PTB1B deletion is known to exert positive effects on leptin sensitivity, decrease in body weight and fat mass, improved glucose homeostasis, and resistance to DIO [79,80,81,82,83]. In contrast, increased PTB1B expression is associated with leptin resistance [84]. PTP1B inhibitors have been developed for use in obese subjects. Among these, thiazolidinedione derivatives have shown their ability to suppress body weight and to improve the circulating lipid profile in HFD mice. Trodusquemine, an allosteric inhibitor of PTBP1B with the ability to traverse the BBB, has also caused considerable reduction in the food intake, fat mass, and body weight in DIO mice [84,85]. Considering the implication of negative regulators of leptin signaling, the use of SOCS3 and PTP1B inhibitors represents an interesting option for use in restoring leptin response. However, there are multiple challenges in the development of these inhibitors for clinical application, with regard to their specificity. SOCS3 and PTP1B are proteins involved in the signaling pathways activated by different agonists that regulate cellular functions, and their inhibition can cause serious alterations; some knock-out models present intrauterine mortality [78,80]. It is, therefore, necessary that these inhibitors specifically act in the neuronal circuits involved in the regulation of body weight.

## 5. Other Potential Therapies

The development of leptin resistance in obesity is also associated with an increase in endoplasmic reticulum (ER) stress in animal models [86]. Chemical chaperones are a group of compounds that have been characterized as agents that increase the functionality of ER and decrease the accumulation and aggregation of misfolded proteins in the ER by reducing ER stress [87]. Four-phenylbutyrate (PBA) and tauroursodeoxycholic acid (TUDCA) are US Food and Drug Administration (FDA)-approved molecules [88,89] that have been used to reduce ER stress at the hypothalamic level, thereby recovering leptin sensitivity in DIO mice by reducing food intake and body weight [86]. Other compounds such as fluvoxamine, a serotonin reuptake inhibitor, and flurbiprofen, a molecule with anti-inflammatory capacity, are able to reduce ER stress and leptin resistance along with causing weight loss in murine models [90,91].

Because various neuropeptides can be delivered into the central nervous system through an intranasal administration route, intranasal leptin might prove an effective treatment approach for obesity. Obese rats receiving leptin intranasally preserve the orexigenic effect of leptin in a manner similar to that observed in non-obese rats [92]. This activates STAT3 phosphorylation in specific parts of the brain and reduces hepatic lipids by increasing the secretion of hepatic triglycerides and decreasing lipogenesis, with a possible therapeutic application for non-alcoholic fatty liver disease (NAFLD) [93]. However, the use of intranasal leptin for the treatment of obese patients presents some challenges that have not yet been overcome, such as high doses of peptidic hormones, variable absorption by the nasal mucosa, and the high price of recombinant leptin.

Irrespective of the mechanism(s) involved in the occurrence of leptin resistance in obese individuals, it is important to note the presence of high concentrations of circulating leptin. This could also be the origin of leptin resistance. High levels of leptin could be responsible for activating the molecular mechanisms underlying leptin resistance, and therefore, a possible strategy could be the reduction of circulating leptin levels to their physiological levels. Previous data obtained, and patented, by our group have shown that the treatment of DIO rats with polyclonal anti-leptin antibody serum caused a reduction in circulating leptin levels, decreased food intake, and caused ~5% loss of body weight.

## 6. Conclusions

The impact of obesity and its effects on human health have increased at very high rates over recent years since the discovery of leptin. Although leptin is the hallmark of obesity and a major appetite suppressant, no effective obesity therapy based on this hormone has been developed. However, research on obesity and metabolism control continues to focus on this interesting hormone because the prevention and treatment of leptin resistance represents one of the greatest challenges in the treatment of obesity. The scientific community has developed experimental animal models in which different leptin-based approaches have shown some success, but no clinically relevant application has been derived to date. Some of these approaches, perhaps most of them, are based on the idea that leptin resistance is caused by impaired leptin transportation across the BBB; however, this idea is yet unclear. Some data implicate another underlying cause, which, if correct, would have a large impact on the clinical application of leptin in the future. New mechanisms and pathways activated by leptin are continuously being discovered, together with the development of new techniques and drug combinations that could improve the effectiveness and safety of leptin. These approaches regenerate the hope of using leptin as an effective treatment for obesity. 

## References

[1] Zhang Y., Proenca R., Maffei M., Barone M., Leopold L., Friedman J.M. (1994). Positional cloning of the mouse obese gene and its human homologue. Nature.

[2] Farooqi I.S., Jebb S.A., Langmack G., Lawrence E., Cheetham C.H., Prentice A.M., Hughes I.A., McCamish M.A., O’Rahilly S. (1999). Effects of recombinant leptin therapy in a child with congenital leptin deficiency. N. Engl. J. Med..

[3] Woods S.C., Schwartz M.W., Baskin D.G., Seeley R.J. (2000). Food intake and the regulation of body weight. Annu. Rev. Psychol..

[4] Considine R.V., Sinha M.K., Heiman M.L., Kriauciunas A., Stephens T.W., Nyce M.R., Ohannesian J.P., Marco C.C., McKee L.J., Bauer T.L. (1996). Serum immunoreactive-leptin concentrations in normal-weight and obese humans. N. Engl. J. Med..

[5] Bjorbaek C., Elmquist J.K., Frantz J.D., Shoelson S.E., Flier J.S. (1998). Identification of SOCS-3 as a potential mediator of central leptin resistance. Mol. Cell.

[6] Chen K., Li F., Li J., Cai H., Strom S., Bisello A., Kelley D.E., Friedman-Einat M., Skibinski G.A., McCrory M.A. (2006). Induction of leptin resistance through direct interaction of C-reactive protein with leptin. Nat. Med..

[7] Kabra D.G., Pfuhlmann K., Garcia-Caceres C., Schriever S.C., Casquero Garcia V., Kebede A.F., Fuente-Martin E., Trivedi C., Heppner K., Uhlenhaut N.H. (2016). Hypothalamic leptin action is mediated by histone deacetylase 5. Nat. Commun..

[8] Rodriguez E.M., Blazquez J.L., Guerra M. (2010). The design of barriers in the hypothalamus allows the median eminence and the arcuate nucleus to enjoy private milieus: The former opens to the portal blood and the latter to the cerebrospinal fluid. Peptides.

[9] Banks W.A., Coon A.B., Robinson S.M., Moinuddin A., Shultz J.M., Nakaoke R., Morley J.E. (2004). Triglycerides induce leptin resistance at the blood-brain barrier. Diabetes.

[10] Banks W.A., DiPalma C.R., Farrell C.L. (1999). Impaired transport of leptin across the blood-brain barrier in obesity. Peptides.

[11] El-Haschimi K., Pierroz D.D., Hileman S.M., Bjorbaek C., Flier J.S. (2000). Two defects contribute to hypothalamic leptin resistance in mice with diet-induced obesity. J. Clin. Investig..

[12] Banks W.A. (2015). Peptides and the blood-brain barrier. Peptides.

[13] Moraes J.C., Coope A., Morari J., Cintra D.E., Roman E.A., Pauli J.R., Romanatto T., Carvalheira J.B., Oliveira A.L., Saad M.J. (2009). High-fat diet induces apoptosis of hypothalamic neurons. PLoS ONE.

[14] Kim D.W., Glendining K.A., Grattan D.R., Jasoni C.L. (2016). Maternal Obesity in the Mouse Compromises the Blood-Brain Barrier in the Arcuate Nucleus of Offspring. Endocrinology.

[15] Van Heek M., Compton D.S., France C.F., Tedesco R.P., Fawzi A.B., Graziano M.P., Sybertz E.J., Strader C.D., Davis H.R. (1997). Diet-induced obese mice develop peripheral, but not central, resistance to leptin. J. Clin. Investig..

[16] Halaas J.L., Boozer C., Blair-West J., Fidahusein N., Denton D.A., Friedman J.M. (1997). Physiological response to long-term peripheral and central leptin infusion in lean and obese mice. Proc. Natl. Acad. Sci. USA.

[17] Caro J.F., Kolaczynski J.W., Nyce M.R., Ohannesian J.P., Opentanova I., Goldman W.H., Lynn R.B., Zhang P.L., Sinha M.K., Considine R.V. (1996). Decreased cerebrospinal-fluid/serum leptin ratio in obesity: A possible mechanism for leptin resistance. Lancet.

[18] Schwartz M.W., Peskind E., Raskind M., Boyko E.J., Porte D. (1996). Cerebrospinal fluid leptin levels: Relationship to plasma levels and to adiposity in humans. Nat. Med..

[19] Faouzi M., Leshan R., Bjornholm M., Hennessey T., Jones J., Munzberg H. (2007). Differential accessibility of circulating leptin to individual hypothalamic sites. Endocrinology.

[20] Banks W.A., Kastin A.J., Huang W., Jaspan J.B., Maness L.M. (1996). Leptin enters the brain by a saturable system independent of insulin. Peptides.

[21] Balland E., Dam J., Langlet F., Caron E., Steculorum S., Messina A., Rasika S., Falluel-Morel A., Anouar Y., Dehouck B. (2014). Hypothalamic tanycytes are an ERK-gated conduit for leptin into the brain. Cell Metab..

[22] Pan W., Hsuchou H., He Y., Sakharkar A., Cain C., Yu C., Kastin A.J. (2008). Astrocyte leptin receptor (ObR) and leptin transport in adult-onset obese mice. Endocrinology.

[23] Hileman S.M., Tornoe J., Flier J.S., Bjorbaek C. (2000). Transcellular transport of leptin by the short leptin receptor isoform ObRa in Madin-Darby Canine Kidney cells. Endocrinology.

[24] Boado R.J., Golden P.L., Levin N., Pardridge W.M. (1998). Up-regulation of blood-brain barrier short-form leptin receptor gene products in rats fed a high fat diet. J. Neurochem..

[25] Bjorbaek C., Elmquist J.K., Michl P., Ahima R.S., van Bueren A., McCall A.L., Flier J.S. (1998). Expression of leptin receptor isoforms in rat brain microvessels. Endocrinology.

[26] Hileman S.M., Pierroz D.D., Masuzaki H., Bjorbaek C., El-Haschimi K., Banks W.A., Flier J.S. (2002). Characterizaton of short isoforms of the leptin receptor in rat cerebral microvessels and of brain uptake of leptin in mouse models of obesity. Endocrinology.

[27] Banks W.A., Niehoff M.L., Martin D., Farrell C.L. (2002). Leptin transport across the blood-brain barrier of the Koletsky rat is not mediated by a product of the leptin receptor gene. Brain Res..

[28] Maness L.M., Banks W.A., Kastin A.J. (2000). Persistence of blood-to-brain transport of leptin in obese leptin-deficient and leptin receptor-deficient mice. Brain Res..

[29] Banks W.A., Clever C.M., Farrell C.L. (2000). Partial saturation and regional variation in the blood-to-brain transport of leptin in normal weight mice. Am. J. Physiol. Endocrinol. Metab..

[30] Schwartz M.W., Woods S.C., Porte D., Seeley R.J., Baskin D.G. (2000). Central nervous system control of food intake. Nature.

[31] Ottaway N., Mahbod P., Rivero B., Norman L.A., Gertler A., D’Alessio D.A., Perez-Tilve D. (2015). Diet-induced obese mice retain endogenous leptin action. Cell Metab..

[32] Pan W.W., Myers M.G. (2018). Leptin and the maintenance of elevated body weight. Nat. Rev. Neurosci..

[33] Kleinert M., Kotzbeck P., Altendorfer-Kroath T., Birngruber T., Tschop M.H., Clemmensen C. (2018). Time-resolved hypothalamic open flow micro-perfusion reveals normal leptin transport across the blood-brain barrier in leptin resistant mice. Mol. Metab..

[34] Harrison L., Schriever S.C., Feuchtinger A., Kyriakou E., Baumann P., Pfuhlmann K., Messias A.C., Walch A., Tschop M.H., Pfluger P.T. (2019). Fluorescent blood-brain barrier tracing shows intact leptin transport in obese mice. Int. J. Obes..

[35] Muller T.D., Sullivan L.M., Habegger K., Yi C.X., Kabra D., Grant E., Ottaway N., Krishna R., Holland J., Hembree J. (2012). Restoration of leptin responsiveness in diet-induced obese mice using an optimized leptin analog in combination with exendin-4 or FGF21. J. Pept. Sci..

[36] Elinav E., Niv-Spector L., Katz M., Price T.O., Ali M., Yacobovitz M., Solomon G., Reicher S., Lynch J.L., Halpern Z. (2009). Pegylated leptin antagonist is a potent orexigenic agent: Preparation and mechanism of activity. Endocrinology.

[37] Hukshorn C.J., Westerterp-Plantenga M.S., Saris W.H. (2003). Pegylated human recombinant leptin (PEG-OB) causes additional weight loss in severely energy-restricted, overweight men. Am. J. Clin. Nutr..

[38] Yi X., Yuan D., Farr S.A., Banks W.A., Poon C.D., Kabanov A.V. (2014). Pluronic modified leptin with increased systemic circulation, brain uptake and efficacy for treatment of obesity. J. Control. Release.

[39] Kovalszky I., Surmacz E., Scolaro L., Cassone M., Ferla R., Sztodola A., Olah J., Hatfield M.P., Lovas S., Otvos L. (2010). Leptin-based glycopeptide induces weight loss and simultaneously restores fertility in animal models. Diabetes Obes. Metab..

[40] Zhang C., Su Z., Zhao B., Qu Q., Tan Y., Cai L., Li X. (2010). Tat-modified leptin is more accessible to hypothalamus through brain-blood barrier with a significant inhibition of body-weight gain in high-fat-diet fed mice. Exp. Clin. Endocrinol. Diabetes.

[41] Price T.O., Farr S.A., Yi X., Vinogradov S., Batrakova E., Banks W.A., Kabanov A.V. (2010). Transport across the blood-brain barrier of pluronic leptin. J. Pharmacol. Exp. Ther..

[42] Morath V., Bolze F., Schlapschy M., Schneider S., Sedlmayer F., Seyfarth K., Klingenspor M., Skerra A. (2015). PASylation of murine leptin leads to extended plasma half-life and enhanced in vivo efficacy. Mol. Pharm..

[43] Castaigne J., Demeule M., Boivin D., Lawrence B., Che C. (2011). Leptin and leptin analog conjugates and uses thereof. U.S. Patent.

[44] Castaigne J., Demeule M., Lawrence B., Boivin D., Che C. (2011). Leptin and leptin analog conjugates and fusion proteins and uses thereof.

[45] Grasso P., Lee D.W., Leinung M.C. (2007). Leptin-related peptides. U.S. Patent.

[46] Grasso P., Rozhavskaya-Arena M., Leinung M.C., Lee D.W. (2001). [D-LEU-4]-OB3, a synthetic leptin agonist, improves hyperglycemic control in C57BL/6Job/ob mice. Regul. Pept..

[47] Leibel R.L., Rosenbaum M., Hirsch J. (1995). Changes in energy expenditure resulting from altered body weight. New Engl. J. Med..

[48] Lecoultre V., Ravussin E., Redman L.M. (2011). The fall in leptin concentration is a major determinant of the metabolic adaptation induced by caloric restriction independently of the changes in leptin circadian rhythms. J. Clin. Endocrinol. Metab..

[49] Doucet E., St Pierre S., Almeras N., Mauriege P., Richard D., Tremblay A. (2000). Changes in energy expenditure and substrate oxidation resulting from weight loss in obese men and women: Is there an important contribution of leptin?. J. Clin. Endocrinol. Metab..

[50] Leibel R.L. (2002). The role of leptin in the control of body weight. Nutr. Rev..

[51] Rosenbaum M., Sy M., Pavlovich K., Leibel R.L., Hirsch J. (2008). Leptin reverses weight loss-induced changes in regional neural activity responses to visual food stimuli. J. Clin. Investig..

[52] Ahima R.S., Prabakaran D., Mantzoros C., Qu D., Lowell B., Maratos-Flier E., Flier J.S. (1996). Role of leptin in the neuroendocrine response to fasting. Nature.

[53] Ravussin Y., Gutman R., Diano S., Shanabrough M., Borok E., Sarman B., Lehmann A., LeDuc C.A., Rosenbaum M., Horvath T.L. (2011). Effects of chronic weight perturbation on energy homeostasis and brain structure in mice. Am. J. Physiol..

[54] Rosenbaum M., Murphy E.M., Heymsfield S.B., Matthews D.E., Leibel R.L. (2002). Low dose leptin administration reverses effects of sustained weight-reduction on energy expenditure and circulating concentrations of thyroid hormones. J. Clin. Endocrinol. Metab..

[55] Aronne L., Fujioka K., Aroda V., Chen K., Halseth A., Kesty N.C., Burns C., Lush C.W., Weyer C. (2007). Progressive reduction in body weight after treatment with the amylin analog pramlintide in obese subjects: A phase 2, randomized, placebo-controlled, dose-escalation study. J. Clin. Endocrinol. Metab..

[56] Ravussin E., Smith S.R., Mitchell J.A., Shringarpure R., Shan K., Maier H., Koda J.E., Weyer C. (2009). Enhanced weight loss with pramlintide/metreleptin: An integrated neurohormonal approach to obesity pharmacotherapy. Obesity.

[57] Trevaskis J.L., Turek V.F., Griffin P.S., Wittmer C., Parkes D.G., Roth J.D. (2010). Multi-hormonal weight loss combinations in diet-induced obese rats: Therapeutic potential of cholecystokinin?. Physiol. Behav..

[58] Clemmensen C., Chabenne J., Finan B., Sullivan L., Fischer K., Kuchler D., Sehrer L., Ograjsek T., Hofmann S.M., Schriever S.C. (2014). GLP-1/glucagon coagonism restores leptin responsiveness in obese mice chronically maintained on an obesogenic diet. Diabetes.

[59] Byun K., Gil S.Y., Namkoong C., Youn B.S., Huang H., Shin M.S., Kang G.M., Kim H.K., Lee B., Kim Y.B. (2014). Clusterin/ApoJ enhances central leptin signaling through Lrp2-mediated endocytosis. EMBO Rep..

[60] Dodd G.T., Decherf S., Loh K., Simonds S.E., Wiede F., Balland E., Merry T.L., Munzberg H., Zhang Z.Y., Kahn B.B. (2015). Leptin and insulin act on POMC neurons to promote the browning of white fat. Cell.

[61] Yan C., Yang Y., Saito K., Xu P., Wang C., Hinton A.O., Yan X., Wu Q., Tong Q., Elmquist J.K. (2015). Meta-chlorophenylpiperazine enhances leptin sensitivity in diet-induced obese mice. Br. J. Pharmacol..

[62] Lundin A., Rondahl H., Walum E., Wilcke M. (2000). Expression and intracellular localization of leptin receptor long isoform-GFP chimera. Biochim. Biophys. Acta.

[63] Belouzard S., Delcroix D., Rouille Y. (2004). Low levels of expression of leptin receptor at the cell surface result from constitutive endocytosis and intracellular retention in the biosynthetic pathway. J. Biol. Chem..

[64] Belouzard S., Rouille Y. (2006). Ubiquitylation of leptin receptor OB-Ra regulates its clathrin-mediated endocytosis. EMBO J..

[65] Couturier C., Sarkis C., Seron K., Belouzard S., Chen P., Lenain A., Corset L., Dam J., Vauthier V., Dubart A. (2007). Silencing of OB-RGRP in mouse hypothalamic arcuate nucleus increases leptin receptor signaling and prevents diet-induced obesity. Proc. Natl. Acad. Sci. USA.

[66] Seo S., Guo D.F., Bugge K., Morgan D.A., Rahmouni K., Sheffield V.C. (2009). Requirement of Bardet-Biedl syndrome proteins for leptin receptor signaling. Hum. Mol. Genet..

[67] Haft C.R., de la Luz Sierra M., Barr V.A., Haft D.H., Taylor S.I. (1998). Identification of a family of sorting nexin molecules and characterization of their association with receptors. Mol. Cell. Biol..

[68] Parks W.T., Frank D.B., Huff C., Renfrew Haft C., Martin J., Meng X., de Caestecker M.P., McNally J.G., Reddi A., Taylor S.I. (2001). Sorting nexin 6, a novel SNX, interacts with the transforming growth factor-beta family of receptor serine-threonine kinases. J. Biol. Chem..

[69] Barr V.A., Lane K., Taylor S.I. (1999). Subcellular localization and internalization of the four human leptin receptor isoforms. J. Biol. Chem..

[70] De Ceuninck L., Wauman J., Masschaele D., Peelman F., Tavernier J. (2013). Reciprocal cross-regulation between RNF41 and USP8 controls cytokine receptor sorting and processing. J. Cell Sci..

[71] Seron K., Couturier C., Belouzard S., Bacart J., Monte D., Corset L., Bocquet O., Dam J., Vauthier V., Lecoeur C. (2011). Endospanins regulate a postinternalization step of the leptin receptor endocytic pathway. J. Biol. Chem..

[72] Vauthier V., Swartz T.D., Chen P., Roujeau C., Pagnon M., Mallet J., Sarkis C., Jockers R., Dam J. (2014). Endospanin 1 silencing in the hypothalamic arcuate nucleus contributes to sustained weight loss of high fat diet obese mice. Gene Ther..

[73] Kim T.H., Choi D.H., Vauthier V., Dam J., Li X., Nam Y.J., Ko Y., Kwon H.J., Shin S.H., Cechetto J. (2014). Anti-obesity phenotypic screening looking to increase OBR cell surface expression. J. Biomol. Screen..

[74] Bjornholm M., Munzberg H., Leshan R.L., Villanueva E.C., Bates S.H., Louis G.W., Jones J.C., Ishida-Takahashi R., Bjorbaek C., Myers M.G. (2007). Mice lacking inhibitory leptin receptor signals are lean with normal endocrine function. J. Clin. Investig..

[75] Reed A.S., Unger E.K., Olofsson L.E., Piper M.L., Myers M.G., Xu A.W. (2010). Functional role of suppressor of cytokine signaling 3 upregulation in hypothalamic leptin resistance and long-term energy homeostasis. Diabetes.

[76] Mori H., Hanada R., Hanada T., Aki D., Mashima R., Nishinakamura H., Torisu T., Chien K.R., Yasukawa H., Yoshimura A. (2004). Socs3 deficiency in the brain elevates leptin sensitivity and confers resistance to diet-induced obesity. Nat. Med..

[77] Howard J.K., Cave B.J., Oksanen L.J., Tzameli I., Bjorbaek C., Flier J.S. (2004). Enhanced leptin sensitivity and attenuation of diet-induced obesity in mice with haploinsufficiency of Socs3. Nat. Med..

[78] Marine J.C., McKay C., Wang D., Topham D.J., Parganas E., Nakajima H., Pendeville H., Yasukawa H., Sasaki A., Yoshimura A. (1999). SOCS3 is essential in the regulation of fetal liver erythropoiesis. Cell.

[79] Elchebly M., Payette P., Michaliszyn E., Cromlish W., Collins S., Loy A.L., Normandin D., Cheng A., Himms-Hagen J., Chan C.C. (1999). Increased insulin sensitivity and obesity resistance in mice lacking the protein tyrosine phosphatase-1B gene. Science.

[80] Klaman L.D., Boss O., Peroni O.D., Kim J.K., Martino J.L., Zabolotny J.M., Moghal N., Lubkin M., Kim Y.B., Sharpe A.H. (2000). Increased energy expenditure, decreased adiposity, and tissue-specific insulin sensitivity in protein-tyrosine phosphatase 1B-deficient mice. Mol. Cell. Biol..

[81] Bence K.K., Delibegovic M., Xue B., Gorgun C.Z., Hotamisligil G.S., Neel B.G., Kahn B.B. (2006). Neuronal PTP1B regulates body weight, adiposity and leptin action. Nat. Med..

[82] Banno R., Zimmer D., De Jonghe B.C., Atienza M., Rak K., Yang W., Bence K.K. (2010). PTP1B and SHP2 in POMC neurons reciprocally regulate energy balance in mice. J. Clin. Investig..

[83] De Jonghe B.C., Hayes M.R., Banno R., Skibicka K.P., Zimmer D.J., Bowen K.A., Leichner T.M., Alhadeff A.L., Kanoski S.E., Cyr N.E. (2011). Deficiency of PTP1B in POMC neurons leads to alterations in energy balance and homeostatic response to cold exposure. Am. J. Physiol. Endocrinol. Metab..

[84] Cho H. (2013). Protein tyrosine phosphatase 1B (PTP1B) and obesity. Vitam. Horm..

[85] Lantz K.A., Hart S.G., Planey S.L., Roitman M.F., Ruiz-White I.A., Wolfe H.R., McLane M.P. (2010). Inhibition of PTP1B by trodusquemine (MSI-1436) causes fat-specific weight loss in diet-induced obese mice. Obesity.

[86] Ozcan L., Ergin A.S., Lu A., Chung J., Sarkar S., Nie D., Myers M.G., Ozcan U. (2009). Endoplasmic reticulum stress plays a central role in development of leptin resistance. Cell Metab..

[87] Perlmutter D.H. (2002). Chemical chaperones: A pharmacological strategy for disorders of protein folding and trafficking. Pediatr. Res..

[88] Chen W.Y., Bailey E.C., McCune S.L., Dong J.Y., Townes T.M. (1997). Reactivation of silenced, virally transduced genes by inhibitors of histone deacetylase. Proc. Natl. Acad. Sci. USA.

[89] Maestri N.E., Brusilow S.W., Clissold D.B., Bassett S.S. (1996). Long-term treatment of girls with ornithine transcarbamylase deficiency. N. Engl. J. Med..

[90] Hosoi T., Yamaguchi R., Noji K., Matsuo S., Baba S., Toyoda K., Suezawa T., Kayano T., Tanaka S., Ozawa K. (2014). Flurbiprofen ameliorated obesity by attenuating leptin resistance induced by endoplasmic reticulum stress. EMBO Mol. Med..

[91] Hosoi T., Baba S., Ozawa K. (2014). Therapeutic potential of flurbiprofen against obesity in mice. Biochem. Biophys. Res. Commun..

[92] Schulz C., Paulus K., Johren O., Lehnert H. (2012). Intranasal leptin reduces appetite and induces weight loss in rats with diet-induced obesity (DIO). Endocrinology.

[93] Hackl M.T., Furnsinn C., Schuh C.M., Krssak M., Carli F., Guerra S., Freudenthaler A., Baumgartner-Parzer S., Helbich T.H., Luger A. (2019). Brain leptin reduces liver lipids by increasing hepatic triglyceride secretion and lowering lipogenesis. Nat. Commun..

